# Evaluation of p53, HoxD10, and E-Cadherin Status in Breast Cancer and Correlation with Histological Grade and Other Prognostic Factors

**DOI:** 10.1155/2014/702527

**Published:** 2014-01-30

**Authors:** Preethi Sekar, Jyotsna Naresh Bharti, Jitendra Singh Nigam, Ankit Sharma, Priyanka Bhatia Soni

**Affiliations:** ^1^Department of Pathology, Deen Dayal Upadhyay Hospital, Hari Nagar, New Delhi 110066, India; ^2^Department of Pathology, Maulana Azad Medical College, Bahadur Shah Zafar Marg, New Delhi 110002, India

## Abstract

*Background*. Study of tumor molecular characteristics is necessary to understand both the risk of breast cancer recurrence and the response to therapy. *Aims*. To evaluate p53, HoxD10, and E-cadherin status in breast cancer and to correlate with histological grade and other prognostic factors. *Material and Methods*. The study was conducted in 60 cases of invasive ductal carcinoma NOS with 20 cases belonging to each grade and evaluation of p53 was done by IHC and that of HoxD10 and E Cadherin status by PCR and correlation was done with histological grade and other prognostic factors. *Result*. p53 expression was seen in 71.67% (43/60) of the tumors. HoxD10 gene was downregulated in 46.67% (28/60) of the tumors. p53 overexpression and lower HoxD10 mRNA levels showed statistically significant association higher histological grade of the tumor (*P* < 0.05). CDH1 gene mutation was seen in 60% (15/25) of the tumors. No significant association was found between p53 expression, HoxD10 gene, CDH1 gene mutation, and other prognostic factors. *Conclusion*. p53 over expression and lower HoxD10 mRNA levels were found to be significantly associated with higher grade tumours. This suggests that p53 and HoxD10 gene play an important tumor suppressor role and the loss of which results in breast cancer progression.

## 1. Introduction

Breast cancer is the most common cause of cancer related mortality in urban Indian women, overtaking the cervical cancer. The incidence is 30–33 per 100000 women in urban India and it is the second commonest cause in rural women [1]. There is a gradual rise in the incidence of breast carcinoma worldwide and it accounts for about 25% of all cancers in women [2]. The study of tumor molecular characteristics has enhanced our understanding of both the risk of breast cancer recurrence and the response to therapy. Some of the genes implicated in breast cancer progression that has been evaluated in this study are the p53, E-cadherin, and HoxD10 gene. p53 gene mutation is a critical step in the development of cancers [3]. The p53 gene is present on chromosome 17p; it regulates entry into S phase of the cell cycle [4, 5] and occurrence of apoptosis in tumor cells [6]. Pharaoh et al. have shown p53 gene mutation in breast cancers to be associated with worse prognosis [7]. The EC gene, CDH1 present on chromosome 16q22.1.2 [8], is a classic tumor suppressor gene [9] and loss of EC results in dedifferentiation and invasion in carcinoma [10]. The majority of the infiltrating lobular carcinomas show a complete loss of EC expression while ductal carcinomas show heterogeneous loss of EC expression which is due to epigenetic transcriptional downregulation [11]. Celebiler et al. showed CDH1 under expression to be associated with histological type, higher tumor grade, stage, and nodal status [12]. The Hox gene network is essential for spatiotemporal cell localisation and for cell to cell signal decoding so as to attain phenotype cell identity [13]. The thoracic Hox genes are involved in breast organogenesis [14], whereas the cervical and lumbosacral Hox genes are involved in progression of breast cancer [15]. The genes indicated in breast cancer progression include HoxD10, B13, and A11 in lumbosacral part and HoxB2, D3, and D4 in cervical part [16]. In the present study of 60 cases of invasive ductal carcinoma NOS, an attempt was made to evaluate the p53 status by IHC, the HoxD10, and E Cadherin status by PCR and to correlate them with histological grade and other prognostic factors.

## 2. Materials and Methods

This is a prospective and retrospective study of randomly selected 60 (20 cases of each grade) cases of invasive ductal carcinoma NOS reported in mastectomy specimens received in the Institute of Pathology, Madras Medical College, during the period between Jan 2010 and Feb 2012. Their representative formalin fixed paraffin embedded tissue samples were subjected to p53 immunohistochemical analysis using supersentive polymer HRP system based on nonbiotin polymeric technology (p53: BioGenex D07 mouse IgG2b) and HoxD10 and E Cadherin gene analysis were done by PCR. An estimation of >10% nuclei distinctly stained with anti-p53 antibody was taken as positive. The detailed history and routine investigations of all the cases were obtained from the archives. For real time polymerase chain reaction for HOXD10, RNA from the samples was purified with RNeasy kit (Qiagen). The real time one step polymerase chain reaction was carried out with Rotor Gene Q system using Rotor gene SYBR green RT PCR kit. HoxD10 gene and *β* actin gene were amplified using the extracted RNA as templates and the following forward and reverse primers: HoxD10 forward (5′CTGTCATGCTCCAGCTCAACCC3′) primers, HoxD10 reverse (5′CTAAGAAAACGTGAGGTTGGCGGTC3′) primers, *β* actin forward (5′CCCCTGGCCAAGGTCATCCATGACAACTTT-3′) primers, and *β* actin reverse (5′GGCCATGAGGTCCACCACCCTGTTGCTGTA-3′) primers. The HoxD10 RNA levels were calculated in the breast cancer samples and the normal breast tissue obtained from autopsy material was used as controls in a relative quantification approach by using a reference gene ACTB1. The relative expression was calculated by deriving the delta CT values for HoxD10 with reference to ACTB1 gene expression. The relative concentration of HoxD10 RNA in tumor samples to that of control samples was calculated as a linear value from the equation 2−^ΔΔCT^. These values were subjected to statistical analysis. For PCR of E-cadherin, a small piece of the fresh tumor tissue and the adjacent normal tissue were collected in sterilized vials containing saline and the DNA was extracted by phenol-chloroform method. The amplified PCR product was then subjected to agarose gel electrophoresis; it was then sequenced and compared with the sequence of DNA isolated from normal tissue for the presence of mutation. Primers used for PCR amplification of E-cadherin exons were as per the sequences described by Berx et al. [17]. Due to economic constraints, CDH1 gene analysis was done only for 25 cases. Correlation between p53, E-cadherin expression, and other clinicopathological prognostic factors was analysed using chi-square test and of HoxD10 correlation with other prognostic factors was done using Kruskal-Wallis test. *P* value <0.05 was taken as critical level of significance.

## 3. Results

**Figure 1:**
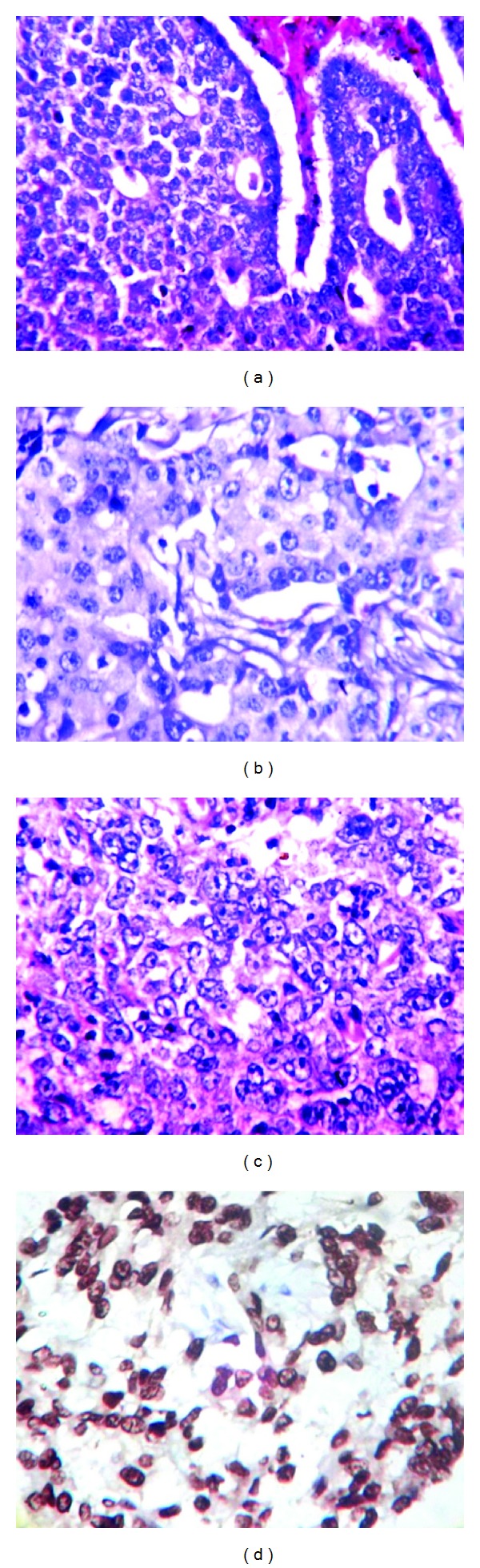
(a) Malignant ductal epithelial cells with mild nuclear pleomorphism and low mitosis-GRADE 1 (H&E ×400). (b) Malignant ductal epithelial cells in sheets, 30% tubules, and mild nuclear pleomorphism-GRADE 2 (H&E ×400). (c) Malignant ductal epithelial cells with no tubules, marked nuclear pleomorphism, and increased mitosis-GRADE 3 (H&E ×400). (d) Invasive ductal carcinoma NOS positive nuclear staining with p53 antibody (BioGenex D07 mouse IgG2b-×400).

20 cases of each grade were randomly selected (Figures 1(a), 1(b), and 1(c)). The age of the patients ranged from 30 to75 years (mean 51.06 years); most of them were in the fifth decade (33.33%). Thirty patients (50%) were premenopausal and thirty (50%) were postmenopausal. The tumor size ranged from 1 to 15 cm (mean 4.78 cm) and most of the tumors (70%) were T2. Thirty-two cases (53.33%) presented with lymph node involvement by metastatic disease. p53 expression was seen in 71.67% (43/60) of the cases. There was a significant correlation between p53 overexpression and higher histological grade of the tumor when calculated for each grade individually (*P* < 0.05) (Table 1). Lower HoxD10 gene mRNA levels were found in 46.67% (28/60) of the tumours and showed statistically significant association with grade III tumours (*P* < 0.001) (Table 2). No statistically significant association was found between p53 expression, HoxD10 gene, and other prognostic factors like age, tumor size, nodal status, skin infiltration, lymphovascular invasion, lymphocytic infiltration, and necrosis.

CDH1 gene mutation was seen in 60% (15/25) of the tumors. However, no association was found between CDH1 gene mutation and grade (Table 3) and other prognostic factors. No statistically significant association was found between p53 expression and CDH1 and HoxD10 gene mutation.

## 4. Discussion

Understanding of the molecular pathways is highly essential as it has important implications in diagnosis, treatment, and prognosis of these patients. The present study showed significant correlation between p53 expression and higher histological grade but correlation with other clinicopathological variables was not significant. Song et al. showed p53 overexpression in 51.6% of the cases and it inversely correlated with lymph node metastasis. The tumor size, histological type, grade, hormone receptor status, and stage of the tumor were not related to the p53 overexpression [18]. Al-Joudi et al. observed p53 expression in 29.6% of the cases and showed significant association with the age and histological grade of the tumor. No association was noted with nodal status, size of tumor, side of the tumor, and ER and PR expression [19]. Aziz and Saleem observed P53 overexpression in 38.3% of the cases and noted significant correlation with patient's age, tumor grade, stage, and size, but no correlation was found with menopausal status and axillary lymph node metastasis [20]. Makiyama et al. observed that there was a significant downregulation of the HoxD10 gene in cancerous tissue compared to noncancerous tissue and no significant correlation with age, menopausal status, tumor size, and serum CEA and CA 125 levels [16]. Ma et al. showed that inhibition of HoxD10 by miR-10b resulted in increased expression of prometastatic gene RHOC leading to tumor invasion and metastasis [21]. Reddy et al. demonstrated that the loss of HoxD10 results in increased motility, invasiveness, anchorage independent growth, and breast cancer progression from low to highly invasive phenotypes [22]. The present study showed significant correlation between HoxD10 gene down regulation and higher histological grade and the correlation with other clinicopathological variables was not significant. Rasti et al. found CDH1 mutation in 41% of the cases and there was a significant correlation between hypermethylation of CDH1 locus and tumor size ≥ 5 cm. The association with other clinicopathological parameters like age, histological type, grade, nodal involvement, and ER and PR status was not found to be significant [23]. Celebiler et al. found CDH1 mutation in 33.9% of the cases and showed significant association with advanced tumor stage, histological type, higher tumor grade, and lymph node metastasis [12]. Shargh et al. noted CDH1 gene mutation in 94% of the cases and found significant association with higher tumor grade, stage, and tumor metastasis [24]. In the present study the correlation between CDH1 gene mutation and other clinicopathological parameters was not found to be significant.

## 5. Conclusion

p53 overexpression and lower HoxD10 mRNA levels were found to be significantly associated with higher grade tumours. This suggests that p53 and HoxD10 gene play an important tumor suppressor role and the loss of which results in breast cancer progression. Although not statistically significant, E Cadherin gene mutation was found to be more common in higher grade tumors. This could be due to the small size of the study sample and investigation in larger series is essential to evaluate its prognostic value. Hence, molecular analysis of breast carcinoma may serve as an important prognostic tool to predict patient outcome and for the development of targeted therapy in this new era of early cancer detection.

## Figures and Tables

**Table 1:** Correlation of grade and p53 expression.

Grade	p53 positive (%)	p53 negative (%)	Pearson chi-square test
Grade 1	13 (65%)	7 (35%)	*P* = 0.18
Grade 2	15 (75%)	5 (25%)	*P* = 0.025
Grade 3	15 (75%)	5 (25%)	*P* = 0.025

**Table 2:** Correlation of grade and HOXD10 gene expression.

Grade	*N*	Mean relative concentration of HoxD10 mRNA	SD	Kruskal-Wallis test
1	20	3.368	5.697	*P* = 0.0001
2	20	6.141	4.898	
3	20	0.054	0.058	

**Table 3:** Correlation of grade and CDH1 gene mutation.

Grade	CDH1 mutation positive (%)	CDH1 mutation negative (%)	Pearson chi-square test
Grade 1	2 (50%)	2 (50%)	*P* = 0.40
Grade 2	8 (53.3%)	7 (46.67%)	
Grade 3	5 (83.3%)	1 (16.67%)	

## References

[1] Agarwal G, Ramakant P (2008). Breast cancer care in India: the current scenario and the challenges for the future. *Breast Care*.

[2] http://globocan.iarc.fr/Pages/fact_sheets_cancer.aspx.

[3] Rivlin N, Brosh R, Oren M, Rotter V (2011). Mutations in the p53 tumor suppressor gene: important milestones at the various steps of tumorigenesis. *Genes and Cancer*.

[4] Norberg T, Klaar S, Kärf G, Nordgren H, Holmberg L, Bergh J (2001). Increased p53 mutation frequency during tumor progression-results from a breast cancer cohort. *Cancer Research*.

[5] Cox LS, Lane DP (1995). Tumour suppressors, kinases and clamps: how p53 regulates the cell cycle in response to DNA damage. *BioEssays*.

[6] Lowe SW, Lin AW (2000). Apoptosis in cancer. *Carcinogenesis*.

[7] Pharaoh PDP, Day NE, Caldas C (1999). Somatic mutations in the p53 gene and prognosis in breast cancer: a meta-analysis. *British Journal of Cancer*.

[8] Berx G, Van Roy F (2001). The E-cadherin/catenin complex: an important gatekeeper in breast cancer tumorigenesis and malignant progression. *Breast Cancer Research*.

[9] Qureshi HS, Linden MO, Divine G, Raju UB (2006). E-cadherin status in breast cancer correlates with histologic type but does not correlate with established prognostic parameters. *American Journal of Clinical Pathology*.

[10] Blick T, Widodo E, Hugo H (2008). Epithelial mesenchymal transition traits in human breast cancer cell lines. *Clinical and Experimental Metastasis*.

[11] Cleton-Jansen A (2002). E-cadherin and loss of heterozygosity at chromosome 16 in breast cancinogenesis: different genetic pathways in ductal and lobular breast cancer?. *Breast Cancer Research*.

[12] Celebiler CA, Sevinc AI, Saydam S (2010). Promoter methylation and expression changes of CDH1 and P16 genes in invasive breast cancer and adjacent normal breast tissue. *Neoplasma*.

[13] Cillo C (2007). Deregulation of the Hox gene network and cancer. *HOX Gene Expression*.

[14] Cantile M, Pettinato G, Procino A, Feliciello I, Cindolo L, Cillo C (2003). In vivo expression of the whole HOX gene network in human breast cancer. *European Journal of Cancer*.

[15] Cantile M, Schiavo G, Terracciano L, Cillo C (2007). The HOX gene network as a potential target for cancer therapy. *Current Cancer Therapy Reviews*.

[16] Makiyama K, Hamada J, Takada M (2005). Aberrant expression of HOX genes in human invasive breast carcinoma. *Oncology Reports*.

[17] Berx G, Cleton-Jansen A, Nollet F (1995). E-cadherin is a tumour/invasion suppressor gene mutated in human lobular breast cancers. *EMBO Journal*.

[18] Song HS, Do YR, Kang SH, Jeong KY, Kim YS (2006). Prognostic significance of immunohistochemical expression of p53 gene product in operable breast cancer. *Cancer Treatment and Research*.

[19] Al-Joudi FS, Iskandar ZA, Rusli J (2008). The expression of p53 in invasive ductal carcinoma of the breast: a study in the North-East States of Malaysia. *Medical Journal of Malaysia*.

[20] Aziz ZW, Saleem HS (2011). p53 in breast carcinoma: an immunohistochemical study. *Annals of the College of Medicine Mosul*.

[21] Ma L, Teruya-Feldstein J, Weinberg RA (2007). Tumour invasion and metastasis initiated by microRNA-10b in breast cancer. *Nature*.

[22] Reddy SD, Ohshiro K, Rayala SK, Kumar R (2008). MicroRNA-7, a homeobox D10 target, inhibits p21-activated kinase 1 and regulates its functions. *Cancer Research*.

[23] Rasti M, Entezam M, Monabati A (2009). Hypermethylation of E-cadherin and estrogen receptor-*α* gene promoter and its association with clinicopathological features of breast cancer in Iranian patients. *Iranian Journal of Medical Sciences*.

[24] Shargh SA, Sakizli M, Farajnia S, Montazer-Saheb S (2011). Evaluation of methylation pattern in promoter region of E-cadherin gene and its relation to tumor grade and stage in breast cancer. *African Journal of Biotechnology*.

